# Embedding gender equality into institutional strategy

**DOI:** 10.1017/gheg.2017.5

**Published:** 2017-05-15

**Authors:** S. Ahmed

**Affiliations:** Wellcome Trust Sanger Institute, Human Genetics, Cambridge, Cambridgeshire, UK

**Keywords:** Diversity, equality, gender, other

## Abstract

The SiS (Sex in Science) Programme on the WGC (Wellcome Genome Campus) was established in 2011. Key participants include the Wellcome Trust Sanger Institute, EMB-EBI (EMBL-European Bioinformatics Institute), Open Targets and Elixir. The key objectives are to catalyse cultural change, develop partnerships, communicate activities and champion our women in science work at a national and international level (http://www.sanger.ac.uk/about/sex-science). In this paper, we highlight some of the many initiatives that have taken place since 2013, to address gender inequality at the highest levels; the challenges we have faced and how we have overcome these, and the future direction of travel.

## Introduction

The attrition of women along the academic career-path is a well-documented phenomenon, and similar to other STEM disciplines. In the Biological Sciences, there are 61% women at the Ph.D. level, which drops to 18% women at the professorial level [1]. Some 55% of Ph.D. students at WTSI are female (students include wet lab scientists, mathematical and computational scientists, these disciplines having different gender profiles nationally – 38% female in the Mathematical Sciences and 17% female in Computer Science [2]). Fifty-five per cent of Post-Doctoral Fellows are women, with women making up only 17% of WTSI senior scientific leadership roles (‘Faculty’). The paucity of women in senior leadership positions is an area that we are committed to addressing. There is also a well-documented pay gap. In the UK, women who work full time earn 17% less than men based on mean hourly earnings (http://webarchive.nationalarchives.gov.uk/+/http://www.hm-treasury.gov.uk/d/robertsreview_introch1.pdf).

The Robert's Report *SET for Success* report (http://webarchive.nationalarchives.gov.uk/20100212235759/http://www.equalities.gov.uk/pdf/297158_WWC_Report_acc.pdf) published in 2002 predicted a shortage of SET graduates. Approximately 820 000 science, engineering and technology (SET) professionals will be required in the UK by 2020 [3]. Recruiting and retaining women in scientific careers can help to meet this shortfall [4, 5]. Furthermore, evidence shows that heterogeneity in groups leads to improved diversity of perspectives, improved organisational performance and efficiency, increased productivity and creativity, better decisions and problem solving. Importantly, it improves the ability to attract and retain the best talent, increases satisfaction and commitment within the workforce, and produces greater flexibility for organisations to respond to changing trends [6, 7].

## Aims and objectives

The Wellcome Genome Campus Sex in Science (SiS) Programme is tasked to ameliorate gender inequality at a strategic level by raising awareness of issues that traditionally face women in science, inspiring women and men at different stages of their scientific careers to progress to more senior levels and driving change in practice and policy. The work is underpinned by WTSI's commitment to the Athena SWAN Charter (http://www.ecu.ac.uk/equality-charters/athena-swan/).

Key goals include:

- Representation – increasing the proportion of female applications for scientific leadership positions and the levels below this that can feed up the pipeline.
- Perceptions – dispelling myths and challenging misconceptions.
- Achievement – ensuring that there are no gender barriers.
- Progression – inspiring women and men at different stages of their scientific careers to progress to senior levels.
- Pay – conducting gender pay gap analysis and working towards abolishing the gender pay gap.

An ambitious action plan underpins our endeavours, which covers areas such as increasing women at the senior scientific levels, greater gender-balance on decision-making committees, ensuring equality of access to promotion and development opportunities, further developing and promoting a women/family friendly culture, awareness and training and increasing the take-up of flexible working/family-friendly policies. We work closely with comparator Biological Sciences Research Institutions, such as the Babraham Institute and Institute of Cancer Research, to share data, good practice and policies and cross-fertilise ideas.

This paper sets out numerous impactful changes that we have made to our policies, practices and way of working, that have transformed us into a more attractive place to work, as evidenced by hard data analysis, staff surveys and individual feedback. We hope that our practical advice can help to propel action within the external genomics landscape.

## Methods

The SiS Programme has clear and visible commitment from the most senior levels of management and equality and diversity is embedded into the fabric of Institutional strategy. Actions on improving the gender balance are highlighted in our current quinquennium strategy (2016–2021) and are central to our goals and identity. This demonstrates commitment at the highest level, as our sustained funding depends on these strategic aims. The programme is supported by the SiS working group, which comprises representation from key staff across the Wellcome Genome Campus, and brings together a synthesis of diverse experiences and perspectives, including input from past and present Directors. Strong leadership and championship serves as a catalyst to embed the spirit of diversity and inclusion across the Campus and enables us to respond to challenges swiftly. The working group meets monthly to review progress and advance initiatives and is supported by a full-time manager and part-time officer. The programme has ring-fenced resource and a substantial number of further activities are funded through the Athena SWAN programme.

We adopt a collaborative approach – communication and staff consultation shape the direction of the Programme. This is achieved through dedicated talks, emails, our intranet, staff surveys and focus groups. The Director updates on progress and promotes related activities, for example, at the annual ‘Sanger Day’. Additionally, we hold regular Town Meetings, where employees and managers can share their views, and also organise discussion panel meetings (e.g. on work–life balance and flexible working) at which information and opinions are shared and sought. As the Campus grows, we ensure that new organisations are aligned with the principles of the programme through our Campus Gateway Policy, and we share and lead expertise and foster a culture of equality.

The backbone of the programme is a series of monthly events, encompassing inspirational talks and interactive workshops. The impact is quantitatively and qualitatively measured, including through event attendance, feedback and staff surveys. We have run approximately 45 events, with an average of 100 attendees (65% women; 35% men). Filmed interviews are showcased on the website – the SiS webpages have been viewed ~10 000 times.

**Fig. 1.**
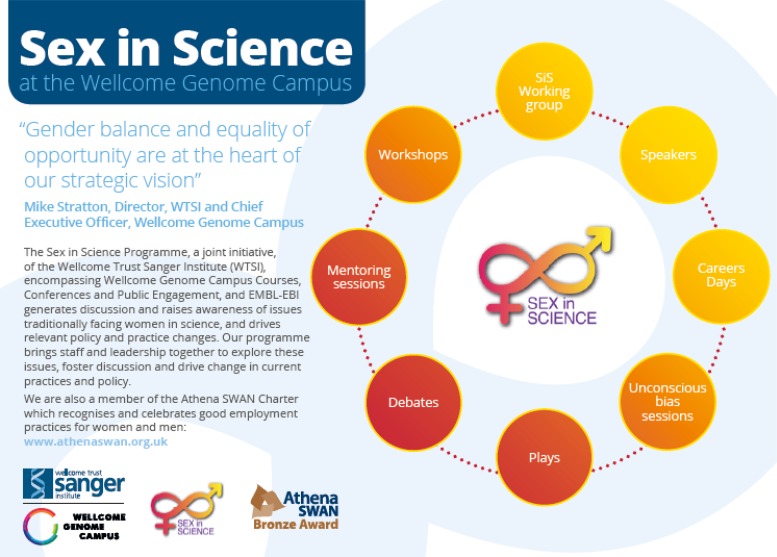
Athena SWAN and SiS information included in the induction pack (front page).

Our commitment to gender equality is woven into the life-cycle of interaction with potential and existing employees. For example, recruitment information on the external website highlights our family-friendly culture and commitment to work–life balance and new employees receive targeted information about the programme in induction packs when they join the Institute (Fig.1).

Changes that have been catalysed by the programme include:

- Specific consideration and outreach by search committees to the potential female applicant pool.
- Mentoring and nurturing budding scientists internally.
- Broadening the eligibility criteria of our decision-making committees, to provide development opportunities to early-career researchers and improve gender balance without overburdening female Faculty. Since introducing these changes in 2014, female representation has increased from 30% to 38%. Along with decreasing the administrative burden of female Faculty, this also allows less senior staff to be exposed to leadership and scientific strategy.
- Educating recruitment panels on unconscious bias. We have run two sessions on unconscious bias for senior managers and two sessions for all staff (>500). We have also run a session for our Board of Management.
- Taking into account career breaks when reviewing job applications.
- Affirmative action statements in recruitment adverts.
- Eradicating explicitly gendered language in job descriptions.
- Developing a women's leadership programme for high-potential future women leaders.
- Promoting internally and externally a women-friendly, family-friendly culture.
- The new Faculty model explicitly cites good citizenship as a criterion for assessment.
- The percentage of female speakers at our GRL organised seminars and conferences has increased to 29%, from 17%, as measured in 2012.

Other positive action initiatives include a returners fellowship (http://www.sanger.ac.uk/about/sex-science/janet-thornton-fellowship), aimed at supporting those who have left science to get back onto the science career path; a workplace nursery, which enables significant savings on the cost of childcare and a carers’ grant, which allows staff to claim back additional childcare costs when attending conferences or meetings. We have distributed the carers’ grant to over 40 members of staff (women and men) and we have made a pledge to meet every request.

“This is brilliant and makes me feel really valued at work.” Female Principal Staff Scientist

We have also introduced a paid leave for carer's policy, which allows staff to take up to an additional 10 paid days leave per year in order to deal with emergency caring responsibilities.

“This is so helpful, many thanks for implementing such a positive change.” Female Informatician

We have updated our parental leave policies (maternity, paternity and shared parental leave) to make them much more favourable for staff. This has had a hugely positive impact on our retention rates – between 2012 and 2015 we have had 88 members of staff go on maternity leave and 100% of these have returned to work.

Moving maternity costs into a central pot has addressed perceived disadvantage to project budgets when staff go on parental leave. As a result of this, the proportion of men taking paternity leave is similar to the proportion of women taking maternity leave and there is a culture within teams and across grades to support paternity leave.

We provide cover when any employee takes maternity or other long-term leave. This can involve allowing other staff to take on additional responsibilities for their own development, and/or the recruitment of short-term staff. If they wish, PDFs can use their ‘Keeping in Touch’ days to oversee key elements of their project. We provide salary extensions to PDFs to extend fixed-term contracts (whether on core or external funding) to take into account maternity or shared parental leave. We also bridge gaps between contracts. Between 2012 and 2015 we extended three of these contracts.

“The terms and conditions of my external grant did not include cover for maternity leave. Sanger covered maternity pay and extended my grant both times I went on maternity leave. This has been extremely important for my career development.” Female Career Development Fellow

We have implemented a favourable student maternity leave policy, which provides students with 6 months on full stipend and intermission for the period of their maternity leave, with their submission deadline extended accordingly (this is explicitly mentioned on the website and during induction). Four female Ph.D. students who were due to submit in the last 3 years have had a period of maternity leave and all four of these students have subsequently submitted their Ph.D. within this extended timeframe.

**Fig. 2.**
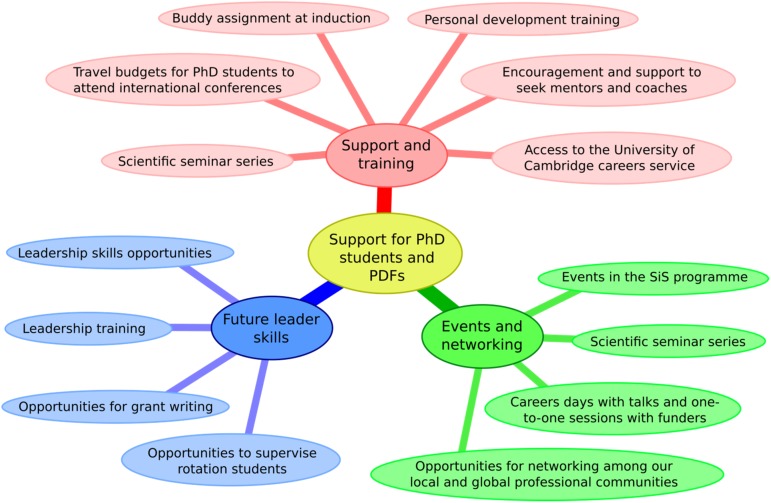
Support for Ph.D. students and PDFs.

We have slightly more women at post-doctoral fellowship level: 54% (*n* = 65) female and 46% (*n* = 55) male. They are represented and supported by the PDF Development Committee, which includes Faculty members alongside PDFs. The PDF model is designed to nurture cohorts of next-generation scientists who will pursue their careers elsewhere at the end of the term and is a fixed-term training contract for 3–5 years. Their tenure is supported by a dedicated PDF training programme (Fig. 2), which includes tailored support to PDFs who want to transition to independence, such as ‘Pathway to Independence’, the new ‘Aspiring Leaders’ programme and the ‘Talented Women's Impact’ Programme. Our ‘Pathway to Independence’ course (in collaboration with the ICR), is a prestigious programme for outstanding PDFs aimed at developing future scientific leaders. Half of all speakers on the programme are female. 100% of delegates rated it “very good” and 90% of the female delegates reported a positive change in their confidence to provide effective leadership for their team.

“I wanted to thank you very much for sending me to the Talented Women's Impact Programme. I really thought they were tremendously helpful and really good. I found it very important for my own growth.” Female PDF.

There is a high success rate of PDFs obtaining independence following attendance of our bespoke leadership courses. Of four women who attended a course in 2013, two have already achieved independence.

Our updated career framework allows PDFs to progress within the Institute into non-Faculty Career Development Fellowship (CDF) positions, a vehicle to progress into scientific leadership. Our first CDF was female.

Faculty reviews encompass academic and institutional achievements, which include strategic fit, research output, good citizenship and mentorship of early-career scientists. Gender equality is embedded into the heart of the Faculty review process, and we have amended our faculty model to allow for flexible extension following maternity leave or other career breaks, e.g. Faculty can extend their review by 1 year per maternity leave. Faculty positions may be held on a part-time employment basis.

Aspiring to become a beacon of good practice and influencing the landscape of science is an ambition we are meeting through leading an equality network for the biosciences sector. This spans both Research Institutes and private sector organisations, and will enable us to benchmark data; share family-friendly policies and good practice and have a collaborative approach to drive positive change.

## Results

The numbers of women in scientific leadership roles has increased from three (2013) to seven (2016). The proportion of women in our Faculty is similar to that of EMBL-European Bioinformatics Institute, which is our closest comparator, and lower than the Institute of Cancer Research and the Babraham Institute (Table 1).

**Table 1.** Faculty benchmarking data from Biological Research Institutes

Institution/data source	Faculty benchmarking statistics
Wellcome Trust Sanger Institute (WTSI)	F: 7 (17%) M: 35 (83%)
EMBL-EBI	F: 2 (17%) M: 10 (83%) Includes Scientific Leaders
Institute of Cancer Research	10 (36%) (F) 18 (64%) (M) Benchmark: Career Faculty; Reader and Professor, Tenure Track Faculty (non-clinicians)
Babraham Institute	7 (27%) (F) 19 (73%) (M) Benchmark: Junior and Senior Group Leaders

We note that female representation (e.g. at the Professorial level in universities) is nationally low at these levels (18% in the Biosciences, 9% in Mathematics and 13% in IT, systems sciences & computer software engineering [1].

Proportions of applications by women to these positions have increased, from 26% to 31% at WTSI. The most recent recruitment round at WTSI saw no difference in the success rate between women and men when they apply for Group Leader positions (statistical significance calculated using Fisher's exact test.) Further progress in this area will be dictated by the frequency and scale of Faculty recruitment. With gender balance at the heart of considerations at the highest level, we are confident that dramatic change will be effected.

**Fig. 3.**
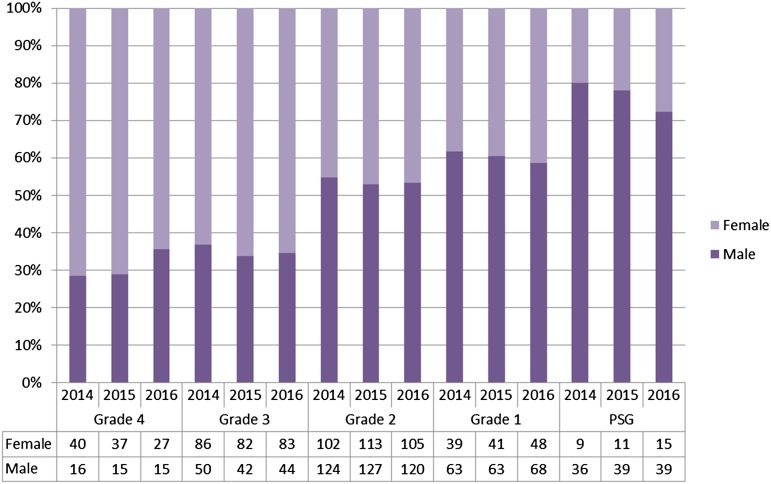
Gender balance of research-related staff across pay grades (excluding Ph.D. students and PDFs) (2014–2016). Note that we do not have any research-related staff on grade 5.

The gender-balance of researchers across higher pay grades has also increased between 2014 and 2016 (Fig. 3). There are 6 grades within our pay framework: Grades 1–5 and a Personal Salary Grade (PSG), which is the highest salary band for staff with strategic responsibilities, including Faculty. PDFs have a dedicated incremental pay scale. Figure 3 shows that the proportion of women has increased at Grade 1 (38% to 41%) and the PSG level (20% to 28%) reflecting good practice in our recruitment and selection processes.

Over the last 3 years more men have applied for PSG roles; however improvements have been made in gender balance here since we were awarded Bronze Award: women and men are shortlisted in approximately equal proportions, and shortlisted women are more successful in being appointed. At grades 1 and 2, women are slightly more likely to be shortlisted, and shortlisted candidates are essentially equally likely to be appointed. At grade 3, there are more women applying, being shortlisted and more women being appointed (statistical significance calculated using Fisher's exact test.)

Similar to our benchmark Institutions, our statistics show attrition at the post-PDF level. To address this we have implemented mechanisms to raise awareness, attract more women applicants to senior positions, inspire early-career women and provide training and development opportunities to nurture rising stars. Since our Bronze Award, 96% (*n* = 50) of women and 86% (*n* = 24) of men Ph.D. students and PDFs agree that the range of training opportunities provided meets their needs (an increase from 75% in 2012).

Our biennial career tracker of Ph.D. students and PDFs shows that our interventions are having a positive impact. The last tracker survey took place in November 2015. Of the 74 respondents 46% (*n* = 34) were female and 49% (*n* = 36) male, while 5% (*n* = 4) did not specify their gender. There were no significant gender differences in age or how long ago they left (statistical significance calculated using Fisher's exact test). The analysis shows that there are no gender differences in the proportions of women and men progressing into leadership positions:

- There were 11 instances of women being named as lead applicants on a grant (17 for men).
- One woman is a CDF and four in scientific leadership positions (12 men).

“My time at the Sanger cemented my ambition to stay in academic science.” Feedback from Female alumna

Both female and male Ph.D. students continue their scientific careers in equitable proportions after they complete their Ph.D.s. Of the 24 women who completed their Ph.D. during the past 3 years, 16 are PDFs, one is an intern in a contract research organisation, one is an MBPh.D. medical student and four are clinicians (one is now a WTSI Faculty member). This is comparable with the data for the 24 men who completed their Ph.D. during the same period. From our 2010 Ph.D. cohort [73% (*n* = 11) female and 27% (*n* = 4) male], three female students have been awarded highly prestigious Wellcome Trust-funded Sir Henry Wellcome Postdoctoral Fellowships. One former female student is a PI in Mexico and another is a clinical lecturer.

Our Coaching and Mentoring scheme (open to all staff) provides a framework through regular workshops and detailed guidance on our intranet. There are 50 mentors and coaches who are provided with full training. Female mentors make up approximately 60% of the network. 65% (*n* = 33) female and 65% (*n* = 18) of male Ph.D. students and PDFs report that they can access a mentor (previously 55% in 2012).

We also encourage women to participate in media and external communications activities to publicise the outcomes of their research. We have significantly increased the number of women quoted in press releases, from 10% in 2012 to 44% in 2015.

As we have proactively promoted and supported flexible working for our staff, the numbers of staff who have requested flexible working has increased by 48% over 3 years – an increase of 42% for women and 86% for men. Managers will often initiate discussions on flexibility with new staff, and we have had positive feedback on this approach. The majority (80%) of employees in our 2016 staff survey [82% (*n* = 268) of women and 77% (*n* = 204) of men] agreed that they are able to balance their work and personal responsibilities. This is an increase from 72% from our 2012 survey.

“Sanger has been very supportive on my return to work following both my children. I feel privileged to be able to advance my career in a job I love under a part-time arrangement. The established nursery onsite also has a very positive impact on my work/life balance. Sanger is a great place to work.” Female Senior Manager

There is a strong culture of informal flexible working, such as daily working patterns that fit around the demands of childcare, the school-run or occasional working from home. The majority of employees in our 2016 survey [82% (*n* = 268) of women and 77% (*n* = 204) of men] agreed that they are able to balance their work and personal responsibilities, an increase from 72% from 2012.

Managers explicitly state in the online recruitment system that roles can be worked flexibly/part-time. Candidates are explicitly asked about career breaks on the application form, and this is taken into account when shortlisting. Guidance and training is available on the full recruitment cycle, including techniques for a fair and systematic approach and highlighting areas where additional consideration needs to be given to avoid discrimination, such as unconscious bias. All of our senior managers (*n* = 108) attended unconscious bias training in July 2015 and we are rolling out regular sessions to all managers.

We have implemented a proactive promotions process, whereby managers actively review eligible staff, focusing on qualitative rather than quantitative outputs, and nominate those they feel are ready to apply for promotion. More women than men were promoted during 2013–2015 (53% women; 47% men). In our 2014 staff survey, 57% (*n* = 141) women and 55% (*n* = 107) men agreed that promotion criteria are fair and transparent. This is a significant increase from 2012, where 35% of staff agreed. We have recently embarked on a review of our promotion processes and criteria and robust criteria and clear guidance for staff and managers has been developed.

At the Faculty level, promotions and renewals are decided according to the prescribed Faculty Model, which details explicit criteria:

- Research output.
- Contribution to education and training.
- Good citizenship, e.g. contribution to internal committees such as ASSAT and SiS.
- Raising the WTSI profile, for example through external committee membership, editorial duties and Public Engagement.
- Promoting an appropriate health and safety culture.

One female faculty member has recently progressed within the Faculty levels. Progression within this group is a key transition point and we are committed to increasing the number of women progressing in seniority.

The number of individuals formally completing an annual appraisal has increased significantly, from 62% in 2013 to 81% in 2015. This is having a positive impact on staff perception of career development: 91% (*n* = 226) women and 86% (*n* = 166) men reported in 2015 that they take ownership of their career development, which is an increase from 36% from 2012.

Our 2014 survey found that increasing proportions of women [81% (*n* = 202)] and men [77% (*n* = 148)] feel that they have a good work-life balance. This is an increase from 72% (2012), demonstrating the positive impact of our policies.

“In my experience the Sanger is a very good place to work. I had great support having children and managed to balance between my family and my work.” Female PDF

## Conclusions

Women are critical to the success of WTSI and addressing gender balance at the highest levels has been prioritised, with this ambition being linked to core funding. Our staff surveys consistently support this sentiment – in our 2016 staff survey, over 92% of the responses on Equality and Diversity were positive, a slight increase from the 2012 staff survey (which was 90%).

Our ambitious action plan for 2016–2019 builds on the positive work we have already achieved. Our Athena SWAN Self-assessment Team (ASSAT) is clearly tasked with analysing data, developing our action plan, and has formal and personal responsibility for delivery and on-going monitoring and evaluation. Key actions to be implemented over the next 3 years include:

- Conducting biennial gender pay audits that go beyond the mandatory gender pay gap reporting. For example, we will analyse gender disaggregated data by job family, job grades and whether there is a part-time pay penalty. We will also examine starting salaries of new staff by gender and ameliorate any gender discrepancies.
- Making a commitment to increase the number of women appointed to Faculty positions. We have a target of increasing the proportion of female applicants from 30% to 50%.
- Ensuring that there is no bias in the recruitment process and all recruitment panel members undergo unconscious bias awareness training.
- Providing support to Faculty and non-Faculty women to progress in seniority, including leadership training, executive coaching and mentoring.
- Improving awareness and increased take-up of flexible working/family-friendly policies. In particular, we are providing further guidance for managers on how to support team members to work flexibly without adversely affecting career trajectories.
- Challenging societal expectations around parental leave, and encouraging and supporting shared parental leave and promoting the compatibility of research and family. Since the shared parental leave legislation came into force in 2015, we have already had three male members of Faculty take-up the enhanced leave provision.
- Striving to influence change in the culture of science and continuing to engage with external organisations to share good practices, thinking and approaches.

Impactful changes that have transformed the Genome Campus into a more attractive place to work have been catalysed by the Sex in Science Programme. Career and leadership opportunities for women have been improved; with enhanced policies and better family friendly on-site facilities. By shining a spotlight on the Institutes’ existing processes and practices and challenging the status quo, we demonstrate that it is possible to drive institutional and cultural change and shift the demographic of existing scientific leadership.

## Staff surveys

Data used in this paper are from the 2012, 2014 and 2016 staff surveys. The response rates were as follows:

- *2016 survey*: overall response rate: 56% (*n* = 606). 54% (*n* = 327) female and 44% (*n* = 265) male respondents.
- *2014 survey*: overall response rate: 47% (*n* = 514). 55% (*n* = 249) female and 42% (*n* = 193) Male respondents.
- *2012 survey*: overall response rate 55% (*n* = 59%). Note that no gender disaggregated data were available.

## References

[1] HESA staff record 2013/14.

[2] Equality in Higher Education – Statistical Report 2015, ECU.

[3] Science and Technology Committee, Seventh Report of Session 2012–13, *Educating tomorrow's engineers: The impact of Government reforms to 14–19 education*, HC 665, para 9.

[4] WSC 74 [Society of Biology] para 1 – written evidence submitted by Society of Biology.

[5] Tapping all our Talents. Women in science, technology, engineering and mathematics: a strategy for Scotland, April 2012, Royal Society of Edinburgh, para 3.

[6] Subeliani D, Tsogas G. Managing diversity in the Netherlands: a case study of Rabobank. International Journal of Human Resource Management 2005; 16: 831–851.

[7] Ozbilgin MF, Tatli A. Mapping out the field of equality and diversity: rise of individualism and voluntarism. Human Relations, 2011; 64: 1229–1253.

